# 2-Fluoro-5-(4-fluoro­phen­yl)pyridine

**DOI:** 10.1107/S1600536812025160

**Published:** 2012-06-13

**Authors:** Fazal Elahi, Muhammad Adeel, M. Nawaz Tahir, Peter Langer, Saeed Ahmad

**Affiliations:** a Department of Chemistry, Gomal University, Dera Ismail Khan, K.P.K, Pakistan; b University of Sargodha, Department of Physics, Sargodha, Pakistan; c Universität Rostock, Institut für Chemie, Abteilung für Organische Chemie, Albert-Einstein-Strasse 3a, 18059 Rostock Department of Chemistry, Germany

## Abstract

In the title compound, C_11_H_7_F_2_N, the fluoro­benzene and the 2-fluoro­pyridine rings are oriented at a dihedral angle of 37.93 (5)°. In the crystal, only van der Waals inter­actions occur.

## Related literature

For a related structure, see: Siddle *et al.* (2010 ▶).
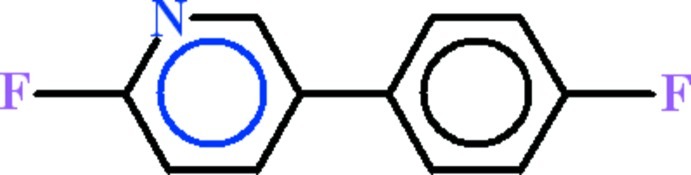


## Experimental

### 

#### Crystal data

C_11_H_7_F_2_N
*M* *_r_* = 191.18
Orthorhombic, 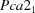

*a* = 20.365 (2) Å
*b* = 3.8303 (3) Å
*c* = 11.4835 (14) Å
*V* = 895.74 (16) Å^3^
*Z* = 4
Mo *K*α radiation
μ = 0.11 mm^−1^
*T* = 296 K
0.26 × 0.20 × 0.18 mm

#### Data collection

Bruker Kappa APEXII CCD diffractometer
Absorption correction: multi-scan (*SADABS*; Bruker, 2005 ▶) *T*_min_ = 0.932, *T*_max_ = 0.950
3601 measured reflections
1489 independent reflections
1162 reflections with *I* > 2σ(*I*)
*R*_int_ = 0.020

#### Refinement

*R*[*F* ^2^ > 2σ(*F* ^2^)] = 0.030
*wR*(*F* ^2^) = 0.072
*S* = 1.03
1489 reflections
128 parameters
1 restraint
H-atom parameters constrained
Δρ_max_ = 0.11 e Å^−3^
Δρ_min_ = −0.09 e Å^−3^

### 

Data collection: *APEX2* (Bruker, 2007 ▶); cell refinement: *SAINT* (Bruker, 2007 ▶); data reduction: *SAINT*; program(s) used to solve structure: *SHELXS97* (Sheldrick, 2008 ▶); program(s) used to refine structure: *SHELXL97* (Sheldrick, 2008 ▶); molecular graphics: *ORTEP-3 for Windows* (Farrugia, 1997 ▶) and *PLATON* (Spek, 2009 ▶); software used to prepare material for publication: *WinGX* (Farrugia, 1999 ▶) and *PLATON*.

## Supplementary Material

Crystal structure: contains datablock(s) global, I. DOI: 10.1107/S1600536812025160/hb6833sup1.cif

Structure factors: contains datablock(s) I. DOI: 10.1107/S1600536812025160/hb6833Isup2.hkl

Supplementary material file. DOI: 10.1107/S1600536812025160/hb6833Isup3.cml

Additional supplementary materials:  crystallographic information; 3D view; checkCIF report

## Figures and Tables

**Fig. 1.**
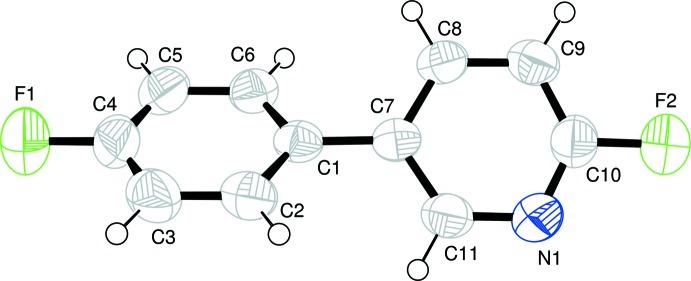
View of the title compound with displacement ellipsoids are drawn at the 50% probability level.

## supplementary crystallographic information

### Comment

The title compound (I), (Fig. 1) is prepared as a precursor and for the study of biological activities.

The crystal structure of 6-fluoro-3-(4-methoxyphenyl)pyridin-2-ol (Siddle *et al.*, 2010) has been published which is related to (I).

In (I) the fluorophenyl A (C1–C6/F1) and the 2-fluoropyridine B (C7—C11/N1/F2) are almost planar with r. m. s. deviations of 0.0025 Å and 0.0071 Å, rspectively. The dihedral angle between A/B is 37.93 (5)°. There does not exist any kind of π-interactions and the molecules must interact due to van Der Waals forces.

### Experimental

To a 6 ml solution of 5-bromo-2-fluoropyridine (0.2 g, 1.136 mmol), 4-fluorophenylboronic acid (0.190 g, 1.36 mmol) in Dioxane and K_3_PO_4_ (0.361 g, 1.5 mmol, in 1 ml H_2_O) was added Pd(PPh_3_)_4_ (1.5 mole %) at 373 K under N_2_ atmosphere. The reaction mixture was refluxed for 8 h. Then 20 ml of distilled water was added. The aqueous layer was extracted three times with EtOAc (3×15 ml). The organic layer was evaporated *in vacuo* and title compound was obtained as a colourless solid. Yield: 0.185 g, 85%. *M*.p. 350–352 K. Crystallization from a saturated CHCl_3_ /CH_3_OH solution gave colorless rods of (I).

### Refinement

The H-atoms were positioned geometrically (C–H = 0.93 Å) and refined as riding with *U*_iso_(H) = *xU*_eq_(C), where *x* = 1.2 for all H-atoms. The absolute structure of the crystal used in this experiment was indeterminate.

### Crystal data

**Table:**

C_11_H_7_F_2_N	*F*(000) = 392
*M**_r_* = 191.18	*D*_x_ = 1.418 Mg m^−^^3^
Orthorhombic, *P**c**a*2_1_	Mo *K*α radiation, λ = 0.71073 Å
Hall symbol: P 2c -2ac	Cell parameters from 1162 reflections
*a* = 20.365 (2) Å	θ = 2.0–25.3°
*b* = 3.8303 (3) Å	µ = 0.11 mm^−^^1^
*c* = 11.4835 (14) Å	*T* = 296 K
*V* = 895.74 (16) Å^3^	Rod, colorless
*Z* = 4	0.26 × 0.20 × 0.18 mm

### Data collection

**Table:**

Bruker Kappa APEXII CCD diffractometer	1489 independent reflections
Radiation source: fine-focus sealed tube	1162 reflections with *I* > 2σ(*I*)
Graphite monochromator	*R*_int_ = 0.020
Detector resolution: 8.10 pixels mm^-1^	θ_max_ = 25.5°, θ_min_ = 2.0°
ω scans	*h* = −21→24
Absorption correction: multi-scan (*SADABS*; Bruker, 2005)	*k* = −2→4
*T*_min_ = 0.932, *T*_max_ = 0.950	*l* = −13→13
3601 measured reflections	

### Refinement

**Table:**

Refinement on *F*^2^	Primary atom site location: structure-invariant direct methods
Least-squares matrix: full	Secondary atom site location: difference Fourier map
*R*[*F*^2^ > 2σ(*F*^2^)] = 0.030	Hydrogen site location: inferred from neighbouring sites
*wR*(*F*^2^) = 0.072	H-atom parameters constrained
*S* = 1.03	*w* = 1/[σ^2^(*F*_o_^2^) + (0.0344*P*)^2^] where *P* = (*F*_o_^2^ + 2*F*_c_^2^)/3
1489 reflections	(Δ/σ)_max_ < 0.001
128 parameters	Δρ_max_ = 0.11 e Å^−^^3^
1 restraint	Δρ_min_ = −0.09 e Å^−^^3^

### Special details

**Table:**

Geometry. Bond distances, angles *etc*. have been calculated using the rounded fractional coordinates. All su's are estimated from the variances of the (full) variance-covariance matrix. The cell e.s.d.'s are taken into account in the estimation of distances, angles and torsion angles
Refinement. Refinement of *F*^2^ against ALL reflections. The weighted *R*-factor *wR* and goodness of fit *S* are based on *F*^2^, conventional *R*-factors *R* are based on *F*, with *F* set to zero for negative *F*^2^. The threshold expression of *F*^2^ > σ(*F*^2^) is used only for calculating *R*-factors(gt) *etc*. and is not relevant to the choice of reflections for refinement. *R*-factors based on *F*^2^ are statistically about twice as large as those based on *F*, and *R*- factors based on ALL data will be even larger.

### Fractional atomic coordinates and isotropic or equivalent isotropic displacement parameters (Å^2^)

**Table:**

	*x*	*y*	*z*	*U*_iso_*/*U*_eq_	
F1	0.16885 (7)	−0.0787 (5)	0.32200 (16)	0.1219 (8)	
F2	0.60621 (6)	0.4445 (4)	0.58901 (13)	0.0984 (6)	
N1	0.50519 (9)	0.2455 (5)	0.62465 (14)	0.0695 (7)	
C1	0.35628 (9)	0.1391 (5)	0.43374 (16)	0.0497 (7)	
C2	0.30232 (10)	0.2088 (6)	0.50519 (18)	0.0635 (9)	
C3	0.23904 (11)	0.1346 (7)	0.4674 (2)	0.0767 (10)	
C4	0.23116 (11)	−0.0048 (7)	0.3597 (2)	0.0785 (11)	
C5	0.28211 (11)	−0.0803 (6)	0.28686 (19)	0.0713 (9)	
C6	0.34494 (10)	−0.0045 (5)	0.32527 (18)	0.0586 (8)	
C7	0.42374 (9)	0.2219 (5)	0.47208 (15)	0.0468 (7)	
C8	0.47047 (9)	0.3501 (6)	0.39465 (17)	0.0560 (8)	
C9	0.53273 (10)	0.4268 (5)	0.43285 (19)	0.0609 (8)	
C10	0.54517 (11)	0.3685 (6)	0.54736 (19)	0.0636 (9)	
C11	0.44444 (10)	0.1735 (5)	0.58562 (18)	0.0603 (8)	
H2	0.30881	0.30545	0.57858	0.0761*	
H3	0.20297	0.17943	0.51471	0.0918*	
H5	0.27490	−0.17911	0.21400	0.0855*	
H6	0.38044	−0.05144	0.27678	0.0703*	
H8	0.45950	0.38384	0.31684	0.0672*	
H9	0.56470	0.51406	0.38289	0.0731*	
H11	0.41423	0.08497	0.63868	0.0723*	

### Atomic displacement parameters (Å^2^)

**Table:**

	*U*^11^	*U*^22^	*U*^33^	*U*^12^	*U*^13^	*U*^23^
F1	0.0663 (9)	0.1613 (16)	0.1380 (14)	−0.0246 (9)	−0.0268 (10)	0.0455 (14)
F2	0.0764 (9)	0.1291 (14)	0.0898 (10)	−0.0286 (8)	−0.0211 (9)	0.0164 (9)
N1	0.0759 (13)	0.0821 (15)	0.0506 (10)	−0.0147 (10)	−0.0079 (10)	0.0035 (9)
C1	0.0565 (12)	0.0413 (12)	0.0512 (11)	0.0046 (9)	0.0032 (9)	0.0085 (9)
C2	0.0662 (15)	0.0613 (17)	0.0629 (13)	0.0067 (11)	0.0106 (11)	0.0089 (10)
C3	0.0576 (15)	0.086 (2)	0.0864 (18)	0.0097 (12)	0.0101 (13)	0.0230 (16)
C4	0.0586 (16)	0.082 (2)	0.095 (2)	−0.0085 (11)	−0.0191 (14)	0.0332 (15)
C5	0.0751 (16)	0.0737 (18)	0.0650 (14)	−0.0054 (12)	−0.0149 (12)	0.0147 (12)
C6	0.0617 (13)	0.0589 (15)	0.0551 (12)	0.0015 (10)	−0.0007 (10)	0.0079 (10)
C7	0.0552 (12)	0.0393 (12)	0.0459 (10)	0.0062 (8)	0.0027 (9)	0.0011 (8)
C8	0.0600 (14)	0.0609 (14)	0.0471 (11)	0.0077 (10)	0.0020 (10)	0.0078 (9)
C9	0.0595 (14)	0.0634 (16)	0.0599 (14)	−0.0003 (10)	0.0094 (10)	0.0089 (10)
C10	0.0596 (15)	0.0635 (16)	0.0678 (14)	−0.0058 (10)	−0.0088 (11)	0.0024 (12)
C11	0.0667 (14)	0.0627 (15)	0.0514 (12)	−0.0080 (10)	0.0065 (10)	0.0050 (10)

### Geometric parameters (Å, º)

**Table:**

F1—C4	1.370 (3)	C7—C8	1.392 (3)
F2—C10	1.363 (3)	C7—C11	1.383 (3)
N1—C10	1.293 (3)	C8—C9	1.374 (3)
N1—C11	1.344 (3)	C9—C10	1.358 (3)
C1—C2	1.397 (3)	C2—H2	0.9300
C1—C6	1.381 (3)	C3—H3	0.9300
C1—C7	1.477 (3)	C5—H5	0.9300
C2—C3	1.389 (3)	C6—H6	0.9300
C3—C4	1.357 (3)	C8—H8	0.9300
C4—C5	1.364 (3)	C9—H9	0.9300
C5—C6	1.384 (3)	C11—H11	0.9300
			
C10—N1—C11	115.17 (18)	F2—C10—C9	118.35 (19)
C2—C1—C6	118.31 (18)	N1—C10—C9	127.4 (2)
C2—C1—C7	121.03 (17)	N1—C11—C7	124.57 (18)
C6—C1—C7	120.66 (17)	C1—C2—H2	120.00
C1—C2—C3	120.47 (19)	C3—C2—H2	120.00
C2—C3—C4	118.4 (2)	C2—C3—H3	121.00
F1—C4—C3	118.6 (2)	C4—C3—H3	121.00
F1—C4—C5	117.8 (2)	C4—C5—H5	121.00
C3—C4—C5	123.6 (2)	C6—C5—H5	121.00
C4—C5—C6	117.6 (2)	C1—C6—H6	119.00
C1—C6—C5	121.70 (19)	C5—C6—H6	119.00
C1—C7—C8	121.42 (16)	C7—C8—H8	120.00
C1—C7—C11	122.40 (17)	C9—C8—H8	120.00
C8—C7—C11	116.18 (17)	C8—C9—H9	122.00
C7—C8—C9	120.17 (18)	C10—C9—H9	122.00
C8—C9—C10	116.52 (19)	N1—C11—H11	118.00
F2—C10—N1	114.27 (19)	C7—C11—H11	118.00
			
C11—N1—C10—F2	179.11 (18)	C2—C3—C4—F1	180.0 (2)
C11—N1—C10—C9	−1.0 (3)	C2—C3—C4—C5	0.8 (4)
C10—N1—C11—C7	0.1 (3)	F1—C4—C5—C6	179.9 (2)
C6—C1—C2—C3	0.0 (3)	C3—C4—C5—C6	−1.0 (4)
C7—C1—C2—C3	179.0 (2)	C4—C5—C6—C1	0.7 (3)
C2—C1—C6—C5	−0.2 (3)	C1—C7—C8—C9	179.62 (19)
C7—C1—C6—C5	−179.19 (19)	C11—C7—C8—C9	−1.1 (3)
C2—C1—C7—C8	−142.1 (2)	C1—C7—C11—N1	−179.87 (18)
C2—C1—C7—C11	38.7 (3)	C8—C7—C11—N1	0.9 (3)
C6—C1—C7—C8	36.9 (3)	C7—C8—C9—C10	0.4 (3)
C6—C1—C7—C11	−142.3 (2)	C8—C9—C10—F2	−179.37 (19)
C1—C2—C3—C4	−0.3 (4)	C8—C9—C10—N1	0.7 (3)
